# Lifestyle Habits Determinants of Health-Related Quality of Life in Moroccan College Students

**DOI:** 10.3390/ijerph20032394

**Published:** 2023-01-29

**Authors:** Doha Achak, Abdelghaffar El-Ammari, Asmaa Azizi, Ibtissam Youlyouz-Marfak, Elmadani Saad, Chakib Nejjari, Abderraouf Hilali, Abdelghafour Marfak

**Affiliations:** 1 Laboratory of Health Sciences and Technologies, Higher Institute of Health Sciences, Hassan First University of Settat, Settat 26000, Morocco; 2 National School of Public Health, Ministry of Health, Rabat 10000, Morocco; 3 International School of Public Health, Mohammed VI University of Health Sciences, Casablanca 82403, Morocco

**Keywords:** university students, health-related quality of life (HRQoL), lifestyle habits determinants

## Abstract

The transition to university is a critical period during which considerable life changes arise. Useful national data to design tailored interventions aimed at promoting health-related quality of life (HRQoL) among Moroccan students are lacking. The present study is aimed at filling this gap by investigating the levels and associated factors of HRQoL among a national sample of Moroccan university students. HRQoL was assessed using the EQ-5D-5L instrument. Data from 2759 university students were collected in a large, cross-sectional, web-based survey. All statistical analyses were conducted using the R software. The EQ-5D-5L findings showed that the majority of students rated level 1 (no problems) and level 2 (slight problems) for the “Mobility”, “Self-Care”, “Usual Activities”, and “Pain/Discomfort” HRQoL dimensions. However, the “Anxiety/Depression” dimension was the exception; more than half (57.1%) of the students were slightly to extremely anxious or depressed. The levels of lifestyle habits were of concern among participants of this study. With respect to sedentary behaviors and physical activity, we found that approximately 80% of participants spent ≥2 h/day on different screen-based sedentary behaviors, and 60% were physically inactive. Lifestyle habits that were found to be associated with HRQoL are sleeping time, physical activity, leisure, hygiene, household activities, homework, and social media time. The multiple regression model explained 93% of the EQ-VAS score variance. The findings could be of great importance for researchers and policymakers interested in promoting health of university students.

## 1. Introduction

Quality of life (QoL) is a broad multidimensional concept which usually includes perceived evaluations of both positive and negative aspects of life, including health, education, work, social network, and economic status [1]. Health-related quality of life (HRQoL), for its part, corresponds to people’s perceived health status over time and the impact of this on QoL [1]. An assessment of HRQoL has increasingly been recognized as a valuable component of public health surveillance and is generally considered a valid indicator of unmet needs and intervention outcomes [1]. That is why HRQoL has become the main outcome in many clinical trials [2]. The transition from high school to university is recognized as a critical period during which considerable individual and contextual changes in almost all dimensions of life take place [3,4]. These changes (e.g., change of residence, increased autonomy and responsibility, and academic and social networks pressure) may result in health-related lifestyle habits that are more likely to persist into adulthood [4]. For example, it was reported that the transition to higher education is an at-risk period for weight gain and unfavorable changes in health behaviors [5].

Hence, the university period may represent the last window of opportunity to promote HRQoL and healthy lifestyle habits, notably among students that have the chance to attend university [4]. Scientific evidence suggests that although university students are often perceived to be healthy, they are exposed to many health risk factors which can compromise their HRQoL and consequently their academic performance and achievement [4,6]. Risk factors correspond with, but are not limited to, socio-demographic factors [7], unhealthy dietary patterns [5,8], physical inactivity and sedentary behaviors [5,9,10,11], smoking [12], high stress [6,12], sleep disorders [13], and a lack of social support [4].

Identifying modifiable determinants of HRQoL among university students is important to develop tailored and effective interventions aimed at promoting student HRQoL and QoL [1]. Research in this field has focused on some age groups (e.g., adolescents and adults) other than university students and has been conducted mainly in developed countries [4,14].

In developing countries such as Morocco, very few studies have focused on measuring the levels and determinants of HRQoL. Some existing studies have focused on the general population or adults with specific health conditions [15,16,17]. In a recent study, poor mental wellbeing was mentioned among Moroccan university students [18]. Hence, there is a need for national studies investigating the levels and determinants of HRQoL in university students. In this study, we aimed at filling this gap by investigating the levels and associated factors of HRQoL among a national sample of Moroccan university students. Filling this gap may result in opportunities to develop actions to improve HRQoL among this specific population.

## 2. Materials and Methods

### 2.1. Study Design and Data Collection

This cross-sectional study was conducted among Moroccan university students aged 18 years and older enrolled in different training programs such as a bachelor’s degree, medicine–dentistry–pharmacy, engineering, and a master’s degree. Data collection was undertaken between 10 and 29 June 2021 using an online self-administered questionnaire in the French language. The study was performed according to the ethical standards of Helsinki’s declaration and approved by the Ethics Committee of Mohamed 6 University of Health Sciences (UM6SS) of Casablanca, Morocco (CERB/UM6SS/24/21—17 May 2021). A summary of the purpose of the study and informed consent were provided to the respondents on the front page of the questionnaire. Participants were assured that their participation is voluntary and anonymous. Access to the questionnaire was allowed only to participants who provided their consent by checking a box, made for this purpose, on the questionnaire. A total of 2759 students agreed to participate in the study and completed the survey consisting of three sections. The first one was related to sociodemographic characteristics (age, gender, educational level, university and/or institution, city of studying, city of residence, and economic level). The second section corresponded to the French version of the EQ-5D-5L instrument in order to measure the students’ HRQoL [19]. The third section included items related to some daily lifestyle habits, which are dietary habits (e.g., number of meals/day and snacking behaviors), physical activity, screen-based sedentary habits, sleep quality, leisure, and hygiene.

Regarding dietary behaviors, the concept of a “meal” refers to an occasion when people sit down and eat, usually at a regular time. Main meals include breakfast, lunch, and dinner. As for the concept of a “snack”, it was defined as eating foods or consuming caloric beverages between regular meals. Having fewer than three meals/day, having at least one snack/day, or having less than three hours between meals were considered unhealthy eating behaviors [20,21]. For physical activity, it is defined as any activity that increases the heart rate and makes the person get out of breath some of the time. Physical activity can be performed in sports, playing with friends, or walking to university. Some examples of physical activity are running, fast walking, biking, dancing, and football. A student with less than 150 min of moderate-intensity physical activity per week was considered physically inactive [22]. With regard to screen time, we distinguished between TV-based screen time and other device-based screen time (i.e., time spent on phone, tablet, laptop, and computer). Furthermore, participants were invited to answer two other questions, one regarding the time spent on phone calls and “SMS”, and the other regarding the time spent on social networks; this was simply to know how much they (i.e., phone calls and “SMS”, and social media) represent within the total screen time. Students must not exceed three hours in screen time (i.e., screen time outside of school or work activities) per day [22]. For sleeping, to be considered as having adequate sleep, a student must report sleeping seven to nine hours of good quality sleep each night. In addition, homework referred to school work that it is usually set by the teacher to do at home. Household activities referred to chores (everyday tasks) performed around the house such as ironing, washing up, and cleaning the house.

### 2.2. Health-Related Quality of Life Measures

The generic EQ-5D-5L questionnaire, used to assess student HRQoL, includes two parts: a descriptive system and a visual analog scale (EQ-VAS) [19]. The descriptive system includes five health dimensions (5D): mobility, self-care, usual activities, pain/discomfort, and anxiety/depression. In our study, we used the EQ-5D-5L version, where five levels are used to represent the degree of the health state severity: no problems (level 1), slight problems (level 2), moderate problems (level 3), severe problems (level 4), or extreme problems (level 5). The participant’s response was converted to a five-digit number describing the health state; for example, 32,415 is a health state equivalent to moderate problems in mobility, slight problems in self-care, severe problems in usual activities, no pain/discomfort, and extreme anxiety/depression. The EQ-VAS, for its part, records the individual’s perception of his/her current health states (scales 0–100, where 0 = the worse imaginable and 100 = the best imaginable). All participants completed both the descriptive system and the EQ-VAS.

### 2.3. Validity of the Daily Lifestyle Habits Questionnaire

#### 2.3.1. Content Validity

To calculate the content validity of the “daily lifestyle habits” questionnaire, two university presidents and three higher institute deans reviewed the relevance of all items of the questionnaire. The relevance was coded on a 4-point Likert scale (1—not relevant, 2—somewhat relevant, 3—relevant, and 4—very relevant). The Item-Content Validity Index (I-CVI) and the Scale-Content Validity Index (S-CVI) were calculated using the criteria of Sangoseni et al. with a threshold of S-CVI >0.78 [23]. The I-CVI was calculated as the proportion of agreement for each item (number of experts giving a 3 or 4 score for that item divided by the number of experts). The S-CVI was calculated as the average of the I-CVIs for the 15 items constituting the “daily lifestyle habits” questionnaire.

#### 2.3.2. Test–Retest Reliability

A sample of approximately 300 participants (20 per item) was targeted. Three days after the first online posting (time 1) of the questionnaire, we collected 340 responses. Seven days after (time 2), the questionnaire was sent by email to all those same participants. Two hundred and ninety-three had completed and sent back the document. The test–retest reliability was evaluated using the Spearman’s rank-order correlation between the responses obtained from the same participants at time 1 and time 2.

#### 2.3.3. Internal Consistency Reliability

Twenty days after the start of the study, we reached 2759 responses. After removing the 340 participants’ data used for the test–retest reliability, the remaining 2419 responses were considered as the final sample. The internal consistency reliability was evaluated based on the Cronbach’s alpha coefficient.

### 2.4. Statistical Analysis

The EQ-5D-5L data were described as percentages obtained for each dimension level. Additionally, percentages were used to describe the lifestyle habits items. A multiple regression was used to assess the relationship between HRQoL and lifestyle variables. The sleep time was used in the regression as the difference between the usual wake-up time and usual bedtime. All statistical analyses were conducted using the R software.

## 3. Results

### 3.1. Participants’ General Characteristics

The study sample consisted of 2419 students, 78.2% were female, and 40.6% were between 18- and 20-years-old. The majority (41.9%) were from the Casablanca–Settat region, 15% from the Rabat–Sale–Kenitra region, 13.0% from Fez–Meknes region, and the remainder were from the other regions (there are 12 regions in our country). Students were enrolled in different levels of training, 42.4% were from a bachelor’s program, 33.7% from medicine–dental–pharmacy studies, and 23.9% were enrolled in either a master’s, doctoral, or engineering program.

Among the 2419 interviewed students, more than 30% were under treatment for one of the following health conditions: allergies, anxiety/depression, anemia, and asthma. Other general characteristics of the participants are given in Table 1.

### 3.2. Health-Related Quality of Life of Students

**Figure 1:**
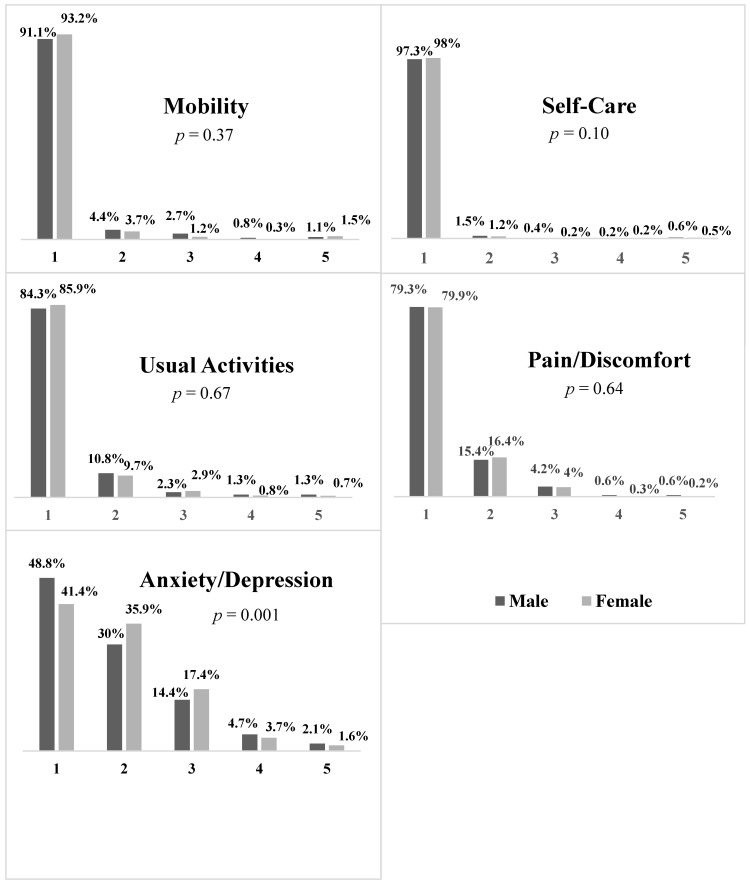
Profile of health-related quality of life among male and female students by levels of severity (1–5). It represents a respondent’s health status according to five dimensions: mobility, self-care, usual activities, pain/discomfort, and anxiety/depression. Within each dimension, there are five severity levels corresponding for mobility to no problem, slight problem, moderate problem, severe problem, and I am unable to walk about. For the self-care dimension, the levels of severity were no problem, slight problem, moderate problem, severe problem, and I am unable to wash or dress myself. Concerning usual activities, the levels corresponding to no problem, slight problem, moderate problem, severe problem, and I am unable to do my usual activities. For pain/discomfort, and anxiety/depression, the levels of severity corresponding to no problem, slight problem, moderate problem, severe problem, I have extreme pain or discomfort for pain/discomfort, and I am extremely anxious or depressed for anxiety/depression.

Regarding the first dimension of the EQ-5D-5L, i.e., “Mobility”, results showed that the majority (92.6%) of students had no problem in walking about, and only 4% had slight problems. For the second dimension, i.e., “Self-Care”, it was found that students had a good autonomy; for instance, 97.8% indicated they “have no or slight problems washing or dressing themselves”. Regarding the third dimension, i.e., “Usual Activities”, 9.8% and 3.5% of students had slight and moderate problems to accomplish their usual activities, respectively. For the fourth dimension, i.e., “Pain/Discomfort”, 78.9% of students had no pain or discomfort. Some students indicated having slight (6.4%) and moderate (4%) pain or discomfort. For the last dimension, i.e., “Anxiety/Depression”, it was the most affected HRQoL domain where 57.1% of students indicated having anxiety/depression. Slight problems were reported by 34.6%, and moderate problems by 16.8% of participants. In addition, a small proportion of students had severe (3.9%) and extreme (1.8%) anxiety. Regarding the VAS scale, the mean VAS score of the participants was 80.35% (±17.63). Furthermore, as shown in the Figure 1, no significant differences were observed between female and male students regarding the first four HRQoL dimensions, that is “Mobility”, “Self-Care”, “Usual Activities”, and “Pain/Discomfort”. However, compared to male students, female students were more anxious/depressed, the fifth dimension of HRQoL (Figure 1).

### 3.3. Daily Lifestyle Habits of Participants

Regarding the validity of the daily lifestyle habits questionnaire, the I-CVIs ranged from 0.8 to 1 and the S-CVI value was 0.91, attesting a very good content validity (CVI > 0.78). The Spearman correlation values for all items were between 0.84 and 0.92, which proves a stronger test–retest reliability. The Cronbach’s alpha coefficient of the questionnaire was equal to 0.72, and the leave-out items ranged from 0.68 to 0.73, indicating a good internal consistency.

The results related to students’ lifestyle habits are shown in Table 2. Regarding eating habits, we observed that the number of meals were 3–4 for 71.6%, and 1–2 snacks for 58.1% of participants. The interval between meals was 3–4 h for 48.9% of students. For sleep habits, the usual bedtime was between 10:00 p.m.–12:00 a.m. for the majority (61.5%) of students. More than 64.0% of students were observed to wake up between 6:00 and 8:00 a.m. The average sleep time was 8.57 h (SD = 1.35), and almost half (49.8%) of students did not take a nap. Furthermore, we found that 61.6% of students were physically inactive. Further, they were less engaged in leisure activities; 51.8% spent less than 2 h. Regarding daily hygiene, the time devoted to personal hygiene was 30 min to 1 h for 49.4%. Approximately 58.2% of students spent less than 1 h doing household activities. A small proportion (8.7%) reported watching TV for at least two hours per day. In addition, 83.5% of students spent at least 2 h per day in screen time (i.e., smartphone, tablet, laptop, and computer). About 54.1% of students spent less than 30 min calling and texting each other, and 63.6% spent between 30 min to 2 h in social media navigation.

### 3.4. Association between Daily Lifestyle Habits and Health-Related Quality of Life

Lifestyle habits associated to student HRQoL were illustrated in Table 3. The multiple regression model explained 93% of the EQ-VAS score variance. HRQoL was positively associated with sleep time (β = +5.25), daily physical activity (β = +3.06), daily leisure (β = +1.87), daily hygiene (β = +2.39), daily household activities (β = +1.71), daily homework (β = +3.61), and daily social media time (β = +2.84). In contrast, students under medical treatment had significantly lower HRQoL (β = −3.82). In addition, daily naptime seemed to negatively impact the VAS scores (β = −1.08).

## 4. Discussion

The main objective of the present study was to investigate the levels and associated factors of HRQoL among a national sample of Moroccan university students. Our results showed that the levels of lifestyle habits were of concern among the participants. In fact, a large proportion of students engaged in unhealthy behaviors, unhealthy eating behaviors (i.e., snacking), sedentary behaviors (i.e., TV-based screen time, as well as other device-based screen time), and physical inactivity. Moreover, most of the lifestyle habits were found to be statistically associated with student HRQoL.

Our findings showed that the majority of students rated level 1 (no problems) and level 2 (slight problems) for the “Mobility”, “Self-Care”, “Usual Activities”, and “Pain/Discomfort” HRQoL dimensions. However, the “Anxiety/Depression” dimension was the exception; more than half (57.1%) of the students were slightly to extremely anxious or depressed. Accordingly, previous research showed that college students, compared to the general population, are at high risk of psychological disorders such as depression and anxiety. In this sense, a study concluded, in a systematic review, that the weighted mean prevalence of depressive disorders among university students was 30.6% which is considerably higher than the rates reported in general populations [24]. This emphasizes the need to identify and manage depression in university settings. Another systematic review showed that stress decreases the QoL of students and through this, the deterioration of various aspects related to physical and mental health [6]; the authors also pointed that factors such as depression and sleeping disorders maximize the aforementioned association [6]. Similar to some previous studies, our results showed that the female students were more anxious/depressed when compared to the male students [6,25]. Several studies mentioned that females are more exposed to mental disorders, specifically depression and anxiety [26].

Regarding sleeping habits, we found that the mean sleep time (8.57 h) was almost similar to that reported by Ge et al. in a sample of college students in Northeast China but it was higher than that found among a national sample of American young adults aged 20–39 years [9,13]. Concerning the association between sleep duration and HRQoL, mixed results have been reported in the literature. In the current study, a positive association was found between sufficient sleep time (≥7 h/weeknight) and HRQoL; such a result has been shown in some studies [9,13]. Given the importance of sleep quality and quantity to physical and mental health, it is necessary to develop interventions aimed at promoting good sleep hygiene and providing college students with alternative skills (e.g., time management skills) to cope with their challenging environment. As we mentioned in the introduction section, the transition from high school to university is a critical period for weight gain [5,21]. Worldwide, a large proportion of university students do not meet the recommended guidelines of dietary, sedentary, and physical activity behaviors [5,9,27]. Results from the current study were not far from that. Indeed, regarding dietary habits, almost one in four of the participants took fewer than three meals and more than two thirds snacked at least once a day. This confirms the fact that unhealthy dietary behaviors are very common among university students [5]. Seeking an understanding of dietary behaviors during the transition from adolescence to young adulthood, Stok et al. found in a literature review that most factors driving this change are at the individual-level (67%) such as food beliefs, time constraints, and taste preferences [27]. Other factors were at the interpersonal-level (e.g., social support), environmental-level (e.g., product characteristics), and policy-level (e.g., market regulations). With respect to sedentary behaviors and physical activity, we found fewer participants watching TV (less than 10% watched ≥2 h/day) but many spent a lot of time on phone calls/SMS, social media, and other device-based screen time (83.5% had a screen time ≥2 h/day), and were physically inactive (more than 60% practiced less than 150 min/week, the threshold to be considered active). These findings are consistent with those from previous studies; for example, Deforche et al. found a decrease in sports participation and in some sedentary behaviors (e.g., TV) during the transition to higher education, while other sedentary behaviors (e.g., internet use and homework) increased. To tackle this issue, the authors suggested that programs to prevent weight gain during university life should therefore already start in high school [5]. Thus, it is evident that the occurrence of a wide range of unhealthy behaviors in students is not accidental; reasons for this are multiple and of a socio-ecological nature (i.e., individual and environmental factors) [27,28]. Individual-level factors include biology, socioeconomic status, and psychological and behavioral characteristics. Environmental-level factors can be separated into social, physical, and macro-level environments. Unhealthy lifestyle habits can lead to various negative consequences on the current and future health of college students, especially those that are called energy-related diseases such as obesity, cardiovascular diseases, and type 2 diabetes [24,29]. Given that, strategies to reduce risky lifestyle habits among college students are needed. To do this, potential partners could use intervention approaches that have been proven to be effective [5,30,31]. Because of socio-cultural and socio-economic differences between countries, it was recommended that national-level data are necessary to inform national decision-makers on the most relevant and tailored policies [32]. Unfortunately, such data on determinants of lifestyle factors are not available in our country making it difficult to develop an effective program; research in this field is thus strongly requested in our country.

Additionally, our results showed that HRQoL was positively associated with some lifestyle habits, namely physical activity, leisure, hygiene, household activities, and homework. Similarly, in a previous study aimed at investigating the association of physical activity, sedentary time, and sleep duration on the HRQoL in Chinese college students, results suggested that PA and sufficient sleep duration may have a positive impact on the HRQoL of college students [9]. The results from another study [33] showed that quality of life significantly and positively correlated with students’ leisure time. Additionally, in a recent systematic review [25] which examined the predictors of QoL among Brazilian medical students, the authors concluded that the main predictors are related to the emotional and physical domains of female students. Accordingly, we recommend including the modifiable factors which were found to be associated with student HRQoL in an intervention program aimed at improving HRQoL among university students [9,34].

## 5. Implications of the Study

Scientific evidence suggests that national-level data are necessary to inform national decision-makers on the most relevant and tailored interventions; this is because of socio-cultural and socio-economic differences between countries. Therefore, the current findings could be useful in developing interventions aimed at promoting healthy lifestyle habits such as dietary behaviors, physical activity, and sleeping among Moroccan university students.

## 6. Limitations of the Study

Because of the cross-sectional nature of this study, it does not allow causal relationships between variables to be established. Furthermore, we were unable to use a validated questionnaire or tool to measure lifestyle variables, and a generated specific questionnaire on “daily lifestyle habits” was used instead. This does not represent a real limitation of our study because, as presented in the Results section, the generated questionnaire showed good content validity, test–retest reliability, and internal consistency. Unfortunately, this questionnaire did not allow for an in-depth study of dietary patterns; in fact, only some aspects (e.g., number of meals and time between meals) were explored, but others (e.g., quality of diet and dietary pattern) were not. Additionally, we measured the association between only a few lifestyle habits and HRQoL; it would have been better to examine this for all potential individual and environmental factors as advocated by some theoretical models, in particular socio-ecological models [35]. Additionally, the study was conducted at a national scale, so some of the registered results could be hard to generalize or replicate due to cultural factors. Finally, it should be mentioned that the majority of participants were female and are enrolled in a health science program.

## 7. Conclusions

Overall, the findings of the present study highlighted that the majority of Moroccan university students showed very good HRQoL; however, some of them were anxious and depressed. Nevertheless, the levels of lifestyle habits were of concern among participants. A large proportion of them engaged in unhealthy behaviors, including snacking behaviors, screen-based sedentary behaviors, and physical inactivity. Most of the lifestyle habits examined were found to be statistically associated with HRQoL (i.e., EQ-VAS score). The findings of the current study highlight the need to design tailored interventions aimed at enhancing healthy lifestyle habits among Moroccan university students. Moreover, given the results on the different dimensions of HRQoL, more focus should be given to interventions related to improving mental health, emotional disorders, and anxiety/depression.

## Figures and Tables

**Table 1:** General characteristics of the study sample.

Variables	*n* (%)
Gender	
Female	1892 (78.2)
Male	527 (21.80)
Age	
18–20	982 (40.6)
21–23	925 (38.2)
≥24	512 (21.2)
Trainings	
Bachelor’s program	1025 (42.4)
Medicine–Dental–Pharmacy program	814 (33.7)
Master’s program	307 (12.7)
PhD program	154 (6.3)
Engineering program	119 (4.9)
Discipline of trainings	
Health sciences	1235 (51.1)
Technical sciences and engineering	452 (18.7)
Economics	212 (8.7)
Human and social sciences	249 (10.3)
Education sciences	73 (3.0)
Others	198 (8.2)
Regions of Morocco	
Casablanca–Settat	1014 (41.91)
Rabat–Sale–Kenitra	363 (15.01)
Fez–Meknes	314 (12.98)
Marrakesh–Safi	264 (10.91)
Oriental	156 (6.45)
Souss–Massa	122 (5.04)
Tangier–Tetouan–Alhouceima	79 (3.27)
Beni Mellal–Khenira	32 (1.32)
Daraa–Tafilalet	26 (1.07)
Guelmim–Oued Noun	19 (0.79)
Laayoune–Sakia Elhamra	19 (0.79)
Dakhla–Oued Ed–Dahab	11 (0.46)
Under medical treatment	
Allergy	237 (9.8)
Anxiety/depression	204 (8.4)
Anemia	215 (8.9)
Asthma	177 (7.3)
None	1586 (65.6)

**Table 2:** Frequencies of lifestyle habits in college students participating in the study.

Variables	*n* (%)	Variables	*n* (%)
Meal per day		Daily hygiene	
1–2	602 (24.9)	Less than 30 min	685 (28.3)
3–4	1731 (71.6)	30 min–1 h	1195 (49.4)
>4	86 (3.6)	1 h–1 h 30	402 (16.6)
Snacking per day		>1 h 30	137 (5.7)
None	653 (27)	Daily household activities	
1–2	1406 (58.1)	<1 h	1408 (58.2)
3–4	314 (13)	1 h–2 h	797 (32.9)
>4	46 (1.9)	2 h–4 h	194 (8)
Interval between Meals (h)		>4 h	20 (0.8)
1–2	397 (16.4)	Daily homework	
3–4	1182 (48.9)	<1 h	369 (15.3)
>4	840 (34.7)	1 h–3 h	1191 (49.2)
Usual bedtime		3 h–5 h	642 (26.5)
8.00 p.m.–10.00 p.m.—22 h	158 (6.5)	>5 h	217 (9)
10.00 p.m.–12.00 a.m.	1487 (61.5)	Time watching TV	
12.00 a.m.–2.00 a.m.	714 (29.5)	Less than 2 h	2208 (91.3)
After 2.00 a.m.	60 (2.5)	2 h–4 h	189 (7.8)
Usual wake-up time		4 h–6 h	15 (0.6)
Before 6.00 a.m.	158 (6.5)	>6 h	7 (0.3)
6.00 a.m.–8.00 a.m.	1554 (64.2)	Daily screen time	
8.00 a.m.–10.00 a.m.	614 (25.4)	Less than 2 h	399 (16.5)
10.00 a.m.–12.00 p.m.	80 (3.3)	2 h–4 h	1129 (46.7)
After 12.00 p.m.	13 (0.5)	4 h–6 h	655 (27.1)
Usual nap length		6 h–8 h	180 (7.4)
None	1204 (49.8)	Daily phone calls and SMS	
Less than 30 min	599 (24.8)	Less than 30 min	1308 (54.1)
30 min–1 h	444 (18.4)	30 min–1 h	671 (27.7)
1 h–2 h	149 (6.2)	1 h–2 h	314 (13)
>2 h	23 (1)	>2 h	126 (5.2)
Daily leisure		Daily social media time	
<1 h	1254 (51.8)	Less than 30 min	445 (18.4)
1 h–3 h	942 (38.9)	30 min–1 h	823 (34)
3 h–5 h	200 (8.3)	1 h–2 h	716(29.6)
>5 h	23 (1)	>2 h	435 (18)
Daily physical activity		>8 h	56 (2.3)
None	791 (32.7)		
Less than 30 min	700 (28.9)		
30 min–1 h	627 (25.9)		
1 h–1 h 30	215 (8.9)		
>1 h 30	86 (3.6)		

**Table 3:** Multiple linear regression of EQ-VAS score on daily lifestyle habits.

Covariates	β [95% CI]	*p*-Value
Under medical treatment	−3.82 [−6.19; −1.46]	0.002
Meals per day	0.003 [−1.77; 1.50]	0.872
Snacking per day	0.7 [−0.39; 1.78]	0.208
Sleep time	5.25 [4.78; 5.72]	<0.0001
Usual nap length	−1.08 [−1.77; −0.39]	0.002
Daily physical activity	3.06 [2.16; 3.96]	<0.0001
Daily leisure	1.87 [0.92; 2.83]	<0.0001
Daily hygiene	2.39 [1.41; 3.37]	<0.0001
Daily household activities	1.71 [0.75; 2.67]	<0.0001
Daily homework	3.61 [2.66; 4.56]	<0.0001
Daily time watching TV	−0.43 [−1.48; 0.63]	0.43
Daily screen time (smartphone tablet, laptop, and computer)	0.19 [−0.82; 1.19]	0.712
Daily social media time	2.84 [1.77; 3.91]	<0.0001
Daily phone calls and SMS	−0.64 [−1.46; 0.18]	0.128

R^2^ = 0.93.

## Data Availability

Data are available upon request by contacting the corresponding author.
